# Supplementary material for: Distinct oligomeric assemblies of STING induced by non-nucleotide agonists
Source: Nat Commun. 2025 Apr 11;16:3440. doi: 10.1038/s41467-025-58641-5 (PMC11992164; doi:10.1038/s41467-025-58641-5)
Supplement: Supplementary file 1 — Supplementary Information [file 41467_2025_58641_MOESM1_ESM.pdf]

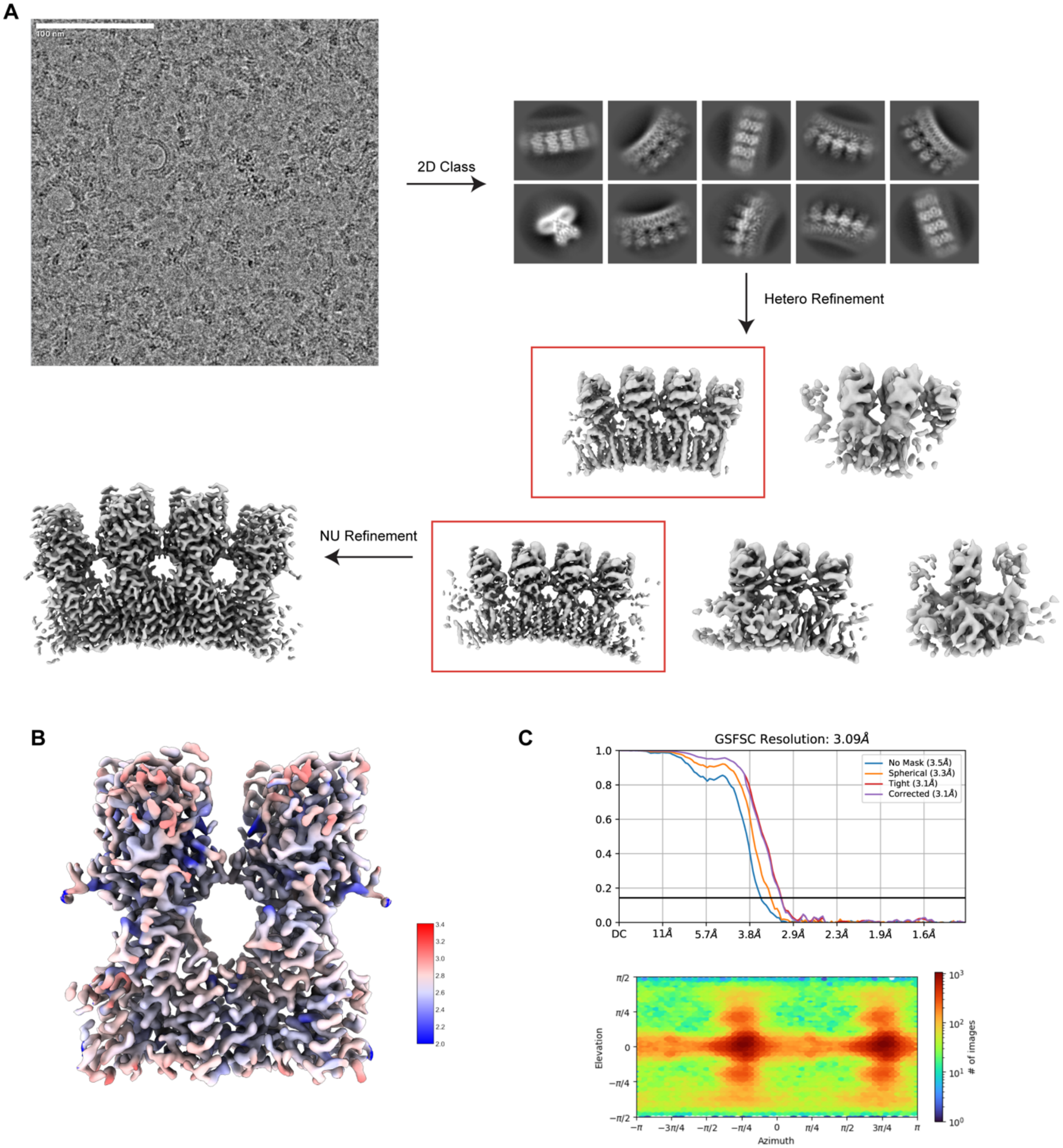

Supplementary Figure 1: SR-717 cryoEM data processing

A. Data processing workflow for SR-717 dataset. B. Local resolution for final SR-717 map. C. FSC curve and angular distribution.

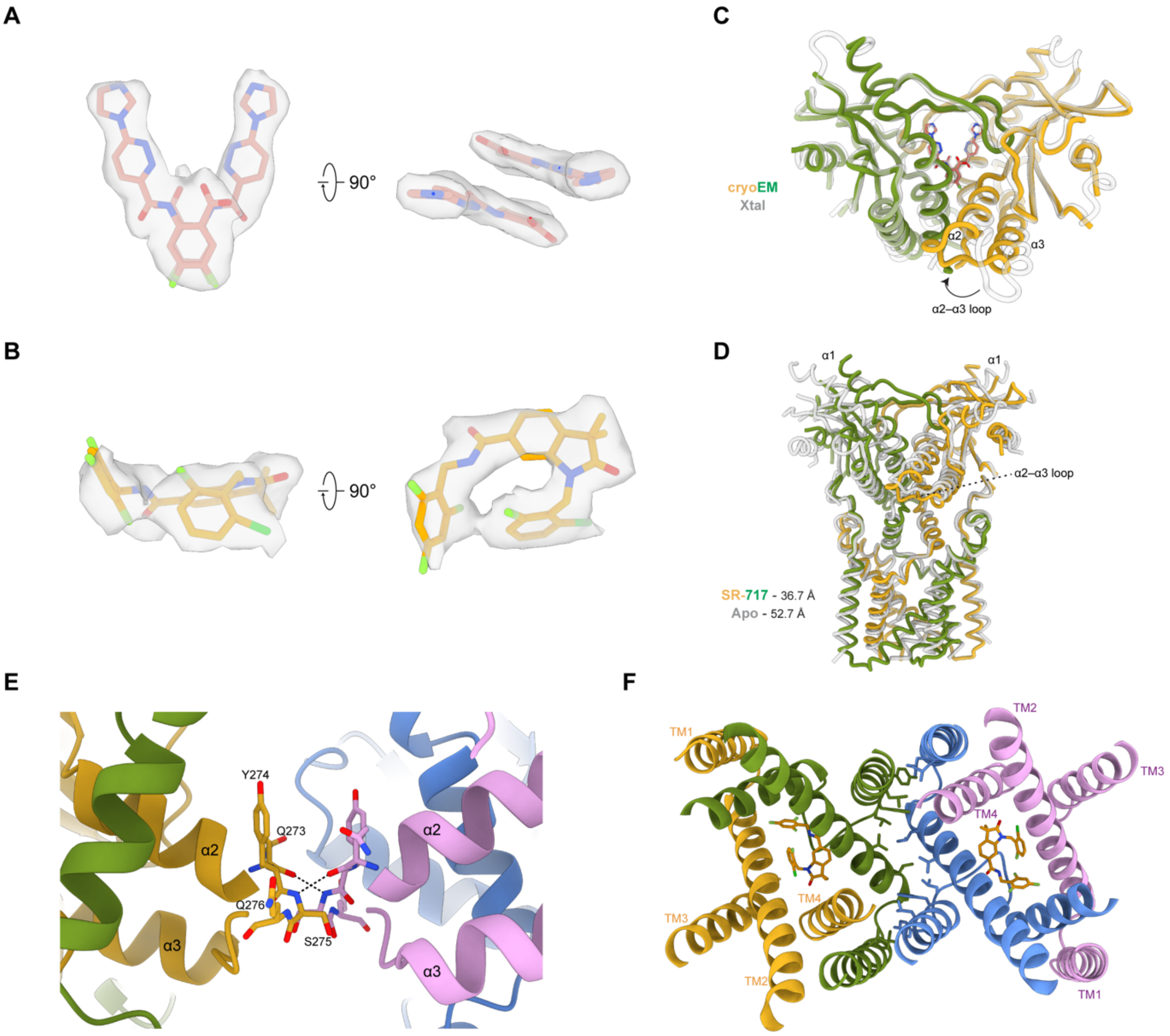

Supplementary Figure 2: SR-717 structure

A. Density for SR-717 ligand. B. Density for C53 ligand. C. Superposition of SR-717 cryoEM structure and LBD crystal structure (PDB ID: 6XNP). D. Superposition of SR-717 cryoEM structure and apo human STING (PDB ID: 6NT5). Distances between α1 helices indicated in bottom left. E. LBD dimeric interaction at the α2- α3 loop. F. Top view of TMD showing dimeric interacting hydrophobic residues as sticks. C53 shown in orange.

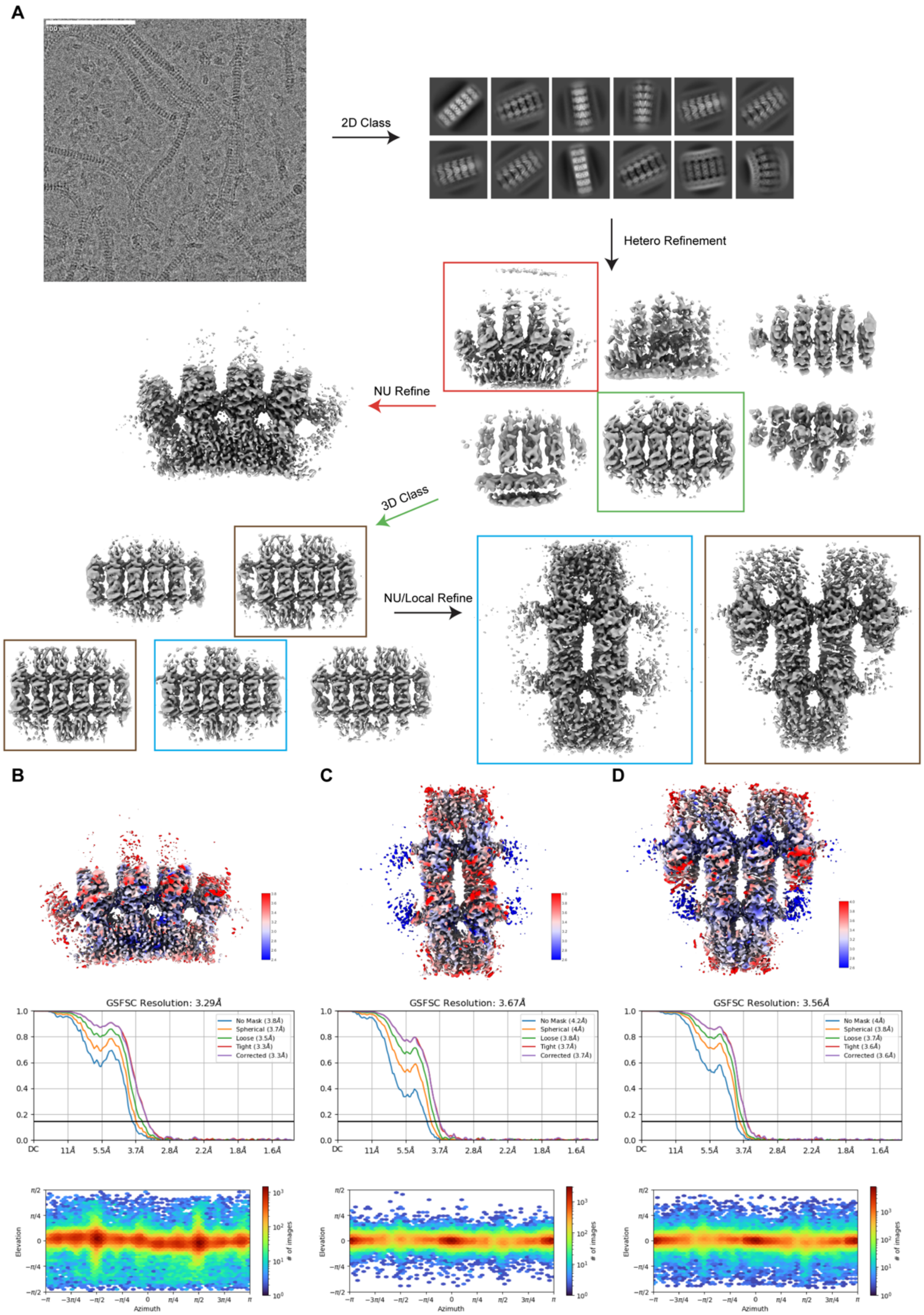

Supplementary Figure 3: diABZI-3 cryoEM data processing

A. Data processing workflow for diABZI-3 dataset. B. Local resolution, FSC, and angular distribution for final curved conformation. C. Local resolution, FSC, and angular distribution for final together conformation. D. Local resolution, FSC, and angular distribution for final apart conformation.

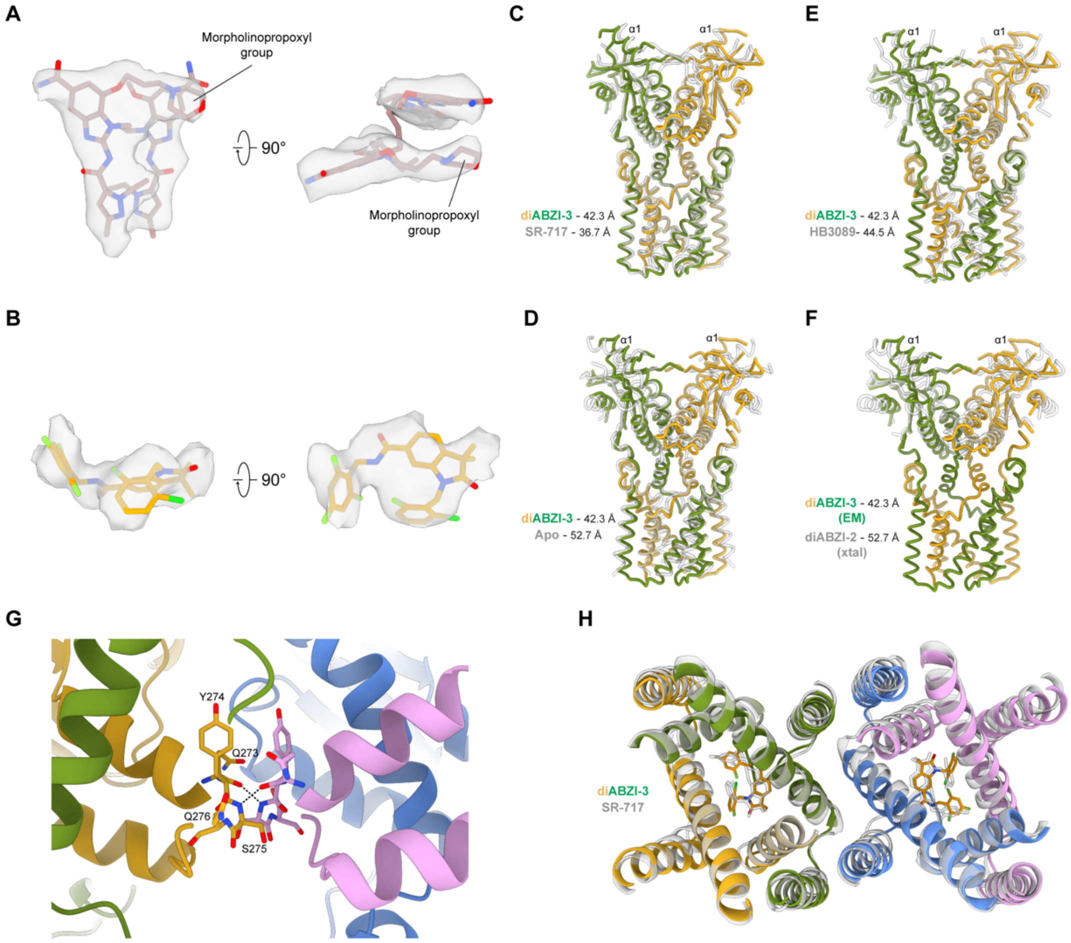

Supplementary Figure 4: diABZI-3 structure

A. Density for diABZI-3 ligand in the together conformation. B. Density for C53 ligand in the together conformation. C. Superposition of diABZI-3 cryoEM structure (together) and SR-717 complex. Distances between α1 helices indicated in bottom left. D. Superposition of diABZI-3 cryoEM structure (together) and apo human STING (PDB ID: 6NT5). E. Superposition of diABZI-3 cryoEM structure and HB3089 complex (PDB ID: 8GT6). F. Superposition of diABZI-3 cryoEM structure and diABZI-2 crystal structure (PDB ID: 6DXL). G. LBD dimeric interaction (together). H. Top view of TMD showing dimeric interacting hydrophobic residues as sticks. C53 shown in orange. SR-717 complex superposed in transparent gray for comparison.

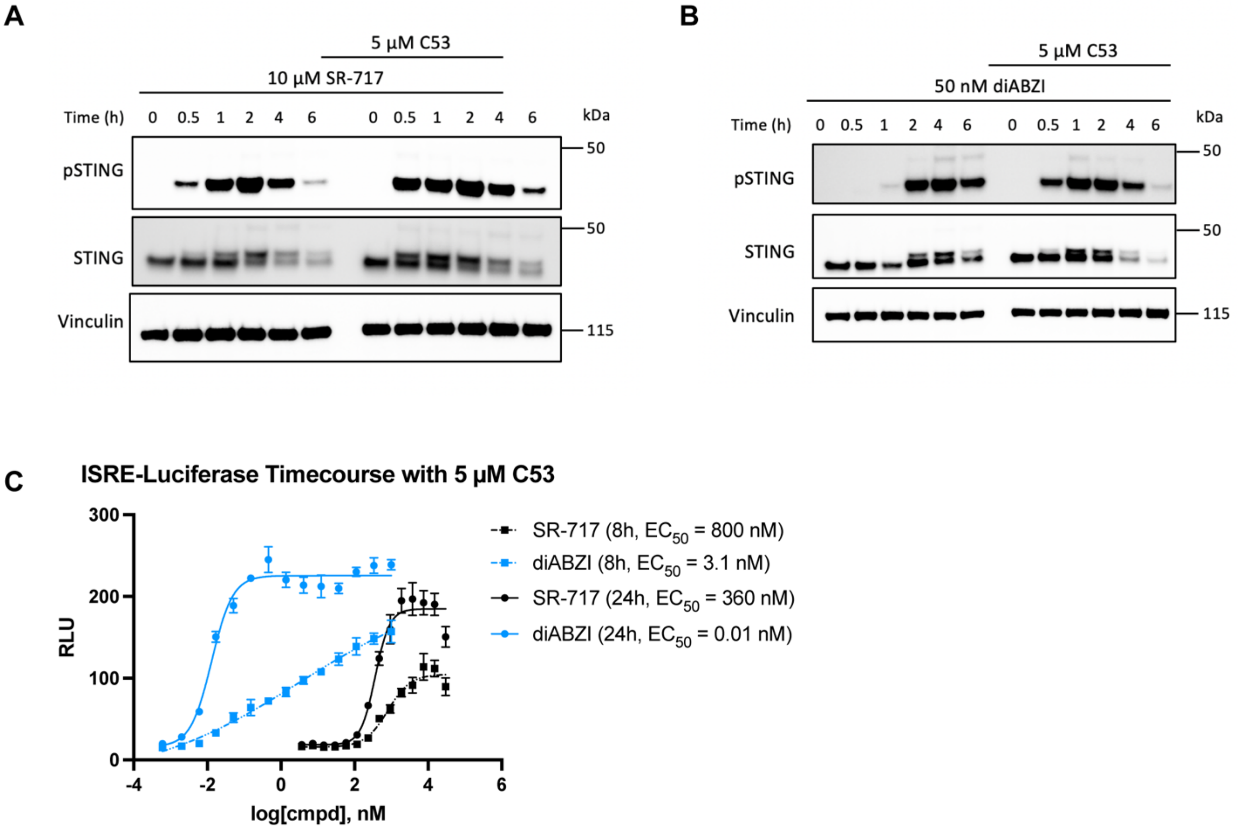

Supplementary Figure 5: Rates of SR-717- and diABZI-3-dependent STING activation in the presence of C53.

A. Timecourse of STING phosphorylation following stimulation with SR-717 in the presence of C53. B. Timecourse of STING phosphorylation following stimulation with diABZI-3 in the presence of C53. C. ISRE-Luciferase dose response for SR-717 and diABZI-3 at 8-and 24-hours post-stimulation in the presence of C53. In (C) data are shown as mean ± SD from n=3 technical replicates. Data presented in (A), (B), and (C) are representative of n=3 independent experiments. Source data are provided as a Source Data file.

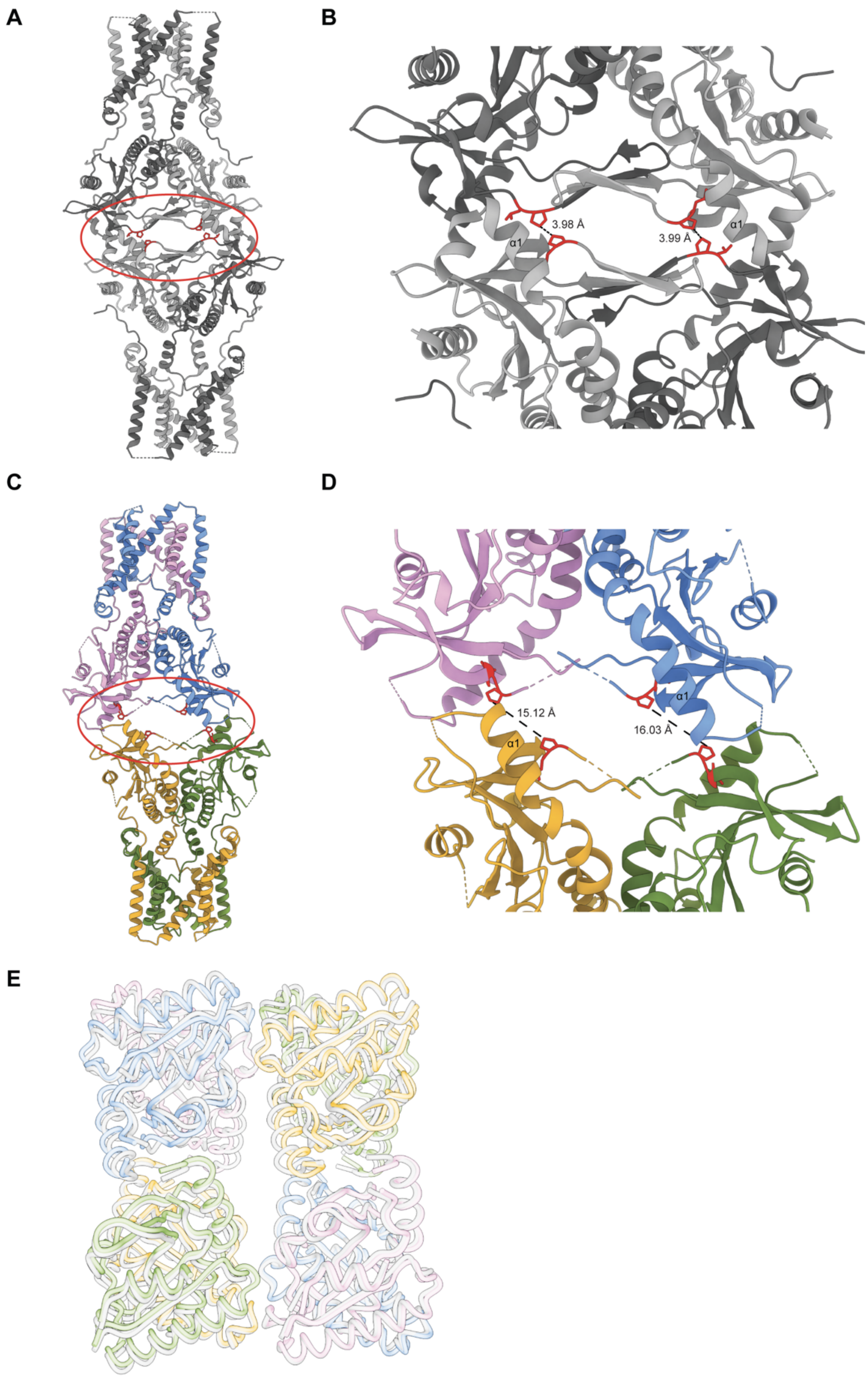

Supplementary Figure 6: Head-to-head interface

A. LP motif in autoinhibited chicken STING (PDB ID: 8IK0). B. Close-up of LP motif in panel A. C. Location of LP motif in diABZI-3 (together) head-to-head interface. D. Close-up of LP motif in panel C. E. Superposition of LBDs in diABZI-3 together (colored) and apart (gray) structures illustrating asymmetry and heterogeneity of the head-to-head interface.

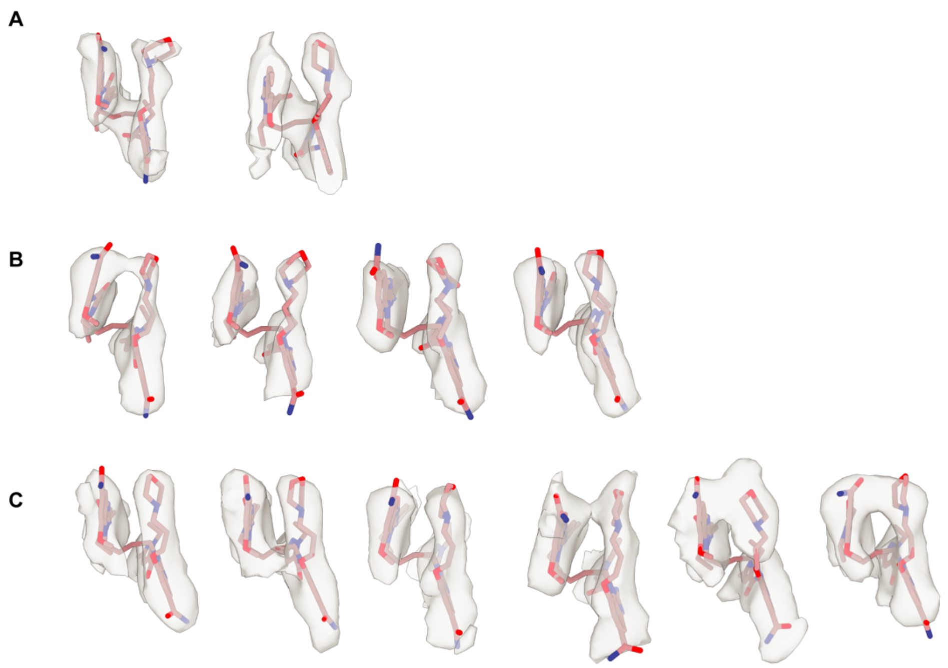

Supplementary Figure 7: diABZI-3 density
A. Density for diABZI-3 ligands in the curved conformation. B. Density for diABZI-3 ligands in the together conformation. C. Density for diABZI-3 ligands in the open conformation

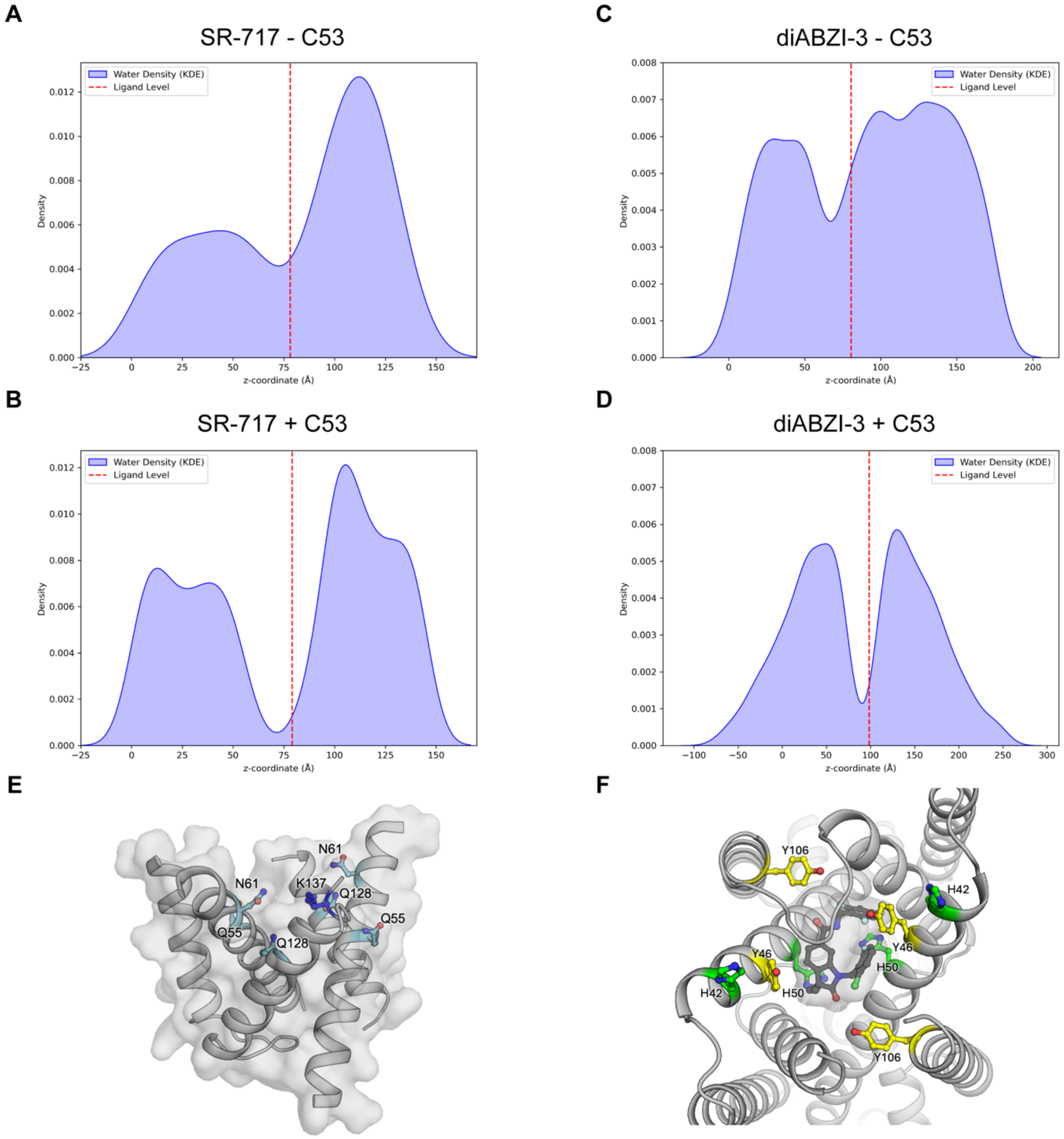

Supplementary Figure 8: Proton pore

A. Kernel density estimation of the water distribution along the pore of the SR-717 complex without C53. The red line indicates the position of the ligand based on the cryo-EM structure. B. Kernel density estimation of the water distribution along the pore of the SR-717 complex with C53. C. Kernel density estimation of the water distribution in the TMD of the diABZI-3 complex without C53. D. Kernel density estimation of the water distribution in the TMD of the diABZI-3 complex with C53. E. Residues involved in the water wire. Hydrophilic residues in blue, non-charged residues in light blue and the

charged residue K137 in dark blue. F. Aromatic residues, namely Tyrosine (yellow) and Histidine (green), crucial for water wires as well as for interactions with C53.

	SR-717 (EMD-45897) (PDB 9CT3)	diABZI Curved (EMD-45898) (PDB 9CT4)	diABZI Apart (EMD-45900) (PDB 9CT6)	diABZI Together (EMDB-45899) (PDB 9CT5)
**Data collection and processing**				
Microscope	TFS Glacios 2		TFS Glacios 2	
Magnification	190,000x		190,000x	
Voltage (kV)	200		200	
Detector	TFS Falcon 4i		TFS Falcon 4i	
Electron exposure ($e^{-}/Å^2$)	45		45	
Defocus range (μm)	-0.6 to -1.5		-0.6 to -1.5	
Pixel size (Å)	0.718		0.718	
Micrographs	4,818		11,729	
Symmetry imposed	C2	C1	C1	C1
Final particle images (no.)	272,478	200,337	410,899	175,203
Map resolution (Å) (FSC threshold 0.143)	3.09	3.29	3.56	3.67
**Refinement**				
Model resolution (Å) (FSC threshold 0.5)	3.19	3.76	4.01	4.00
Map sharpening *B* factor ($Å^2$)	-101.6	-83.4	-100.6	-98.0
Model composition				
Non-hydrogen atoms	10,138	9,938	29,814	19,876
Protein residues	1266	1234	3702	2468
Ligands	6	4	12	8
Map CC	0.83	0.70	0.62	0.72
R.m.s. deviations				
Bond lengths (Å)	0.003	0.003	0.002	0.003
Bond angles (°)	0.562	0.605	0.476	0.520
Validation				
MolProbity score	1.43	1.50	1.42	1.43
Clashscore	7.81	9.56	7.59	7.92
Poor rotamers (%)	0.00	0.00	0.00	0.00
Ramachandran plot				
Favored (%)	98.30	98.00	98.27	98.50
Allowed (%)	1.70	2.00	1.73	1.50
Disallowed (%)	0.00	0.00	0.00	0.00

Supplementary Table 1: Cryo-EM data collection, refinement and validation statistics
