# Supplementary material for: Distinct oligomeric assemblies of STING induced by non-nucleotide agonists
Source: Nat Commun. 2025 Apr 11;16:3440. doi: 10.1038/s41467-025-58641-5 (PMC11992164; doi:10.1038/s41467-025-58641-5)
Supplement: Supplementary file 2 — Reporting Summary [file 41467_2025_58641_MOESM2_ESM.pdf]

nature portfolio

Corresponding author(s): Andrew B Ward, Luke L Lairson

Last updated by author(s): Mar 13, 2025

Reporting Summary

Nature Portfolio wishes to improve the reproducibility of the work that we publish. This form provides structure for consistency and transparency in reporting. For further information on Nature Portfolio policies, see our Editorial Policies and the Editorial Policy Checklist.

Statistics

For all statistical analyses, confirm that the following items are present in the figure legend, table legend, main text, or Methods section.

n/a	Confirmed	
☐	☒	The exact sample size (n) for each experimental group/condition, given as a discrete number and unit of measurement
☐	☒	A statement on whether measurements were taken from distinct samples or whether the same sample was measured repeatedly
☒	☐	The statistical test(s) used AND whether they are one- or two-sided<br>*Only common tests should be described solely by name; describe more complex techniques in the Methods section.*
☒	☐	A description of all covariates tested
☒	☐	A description of any assumptions or corrections, such as tests of normality and adjustment for multiple comparisons
☐	☒	A full description of the statistical parameters including central tendency (e.g. means) or other basic estimates (e.g. regression coefficient) AND variation (e.g. standard deviation) or associated estimates of uncertainty (e.g. confidence intervals)
☒	☐	For null hypothesis testing, the test statistic (e.g. F, t, r) with confidence intervals, effect sizes, degrees of freedom and P value noted<br>*Give P values as exact values whenever suitable.*
☒	☐	For Bayesian analysis, information on the choice of priors and Markov chain Monte Carlo settings
☒	☐	For hierarchical and complex designs, identification of the appropriate level for tests and full reporting of outcomes
☒	☐	Estimates of effect sizes (e.g. Cohen's d, Pearson's r), indicating how they were calculated

Our web collection on statistics for biologists contains articles on many of the points above.

Software and code

Policy information about availability of computer code

Data collection	EPU software was used for automated collection of EM data (ThermoFisher)
Data analysis	Micrographs were aligned using Patch Motion Correction and CTF estimation was done by Patch CTF in CryoSPARC Live50. Micrographs with CTF fits worse than 8 Å were discarded. All following processing was done using CryoSPARC. The final maps were resolved using Non-Uniform Refinement, Global and Local CTF Refinement, and Local Refinement. PDB 7SII was used as an initial model for all STING structures. The model was fit into experimental maps using UCSF ChimeraX52 and morphed into the density using Phenix53. The models were manually adjusted using Coot54,55 and further refined using real-space refinement in Phenix. Structural figures were made using UCSF ChimeraX and Pymol.

For manuscripts utilizing custom algorithms or software that are central to the research but not yet described in published literature, software must be made available to editors and reviewers. We strongly encourage code deposition in a community repository (e.g. GitHub). See the Nature Portfolio guidelines for submitting code & software for further information.

Data

Policy information about availability of data

All manuscripts must include a data availability statement. This statement should provide the following information, where applicable:

- Accession codes, unique identifiers, or web links for publicly available datasets
- A description of any restrictions on data availability
- For clinical datasets or third party data, please ensure that the statement adheres to our policy

Data availability:

The atomic models and cryo-EM density maps generated in this study have been deposited to the PDB and EMDB respectively. The accession numbers are 9CT3 [https://doi.org/10.2210/pdb9CT3/pdb] and EMD-45897 [https://www.ebi.ac.uk/emdb/EMD-45897] (SR-717), 9CT4 [https://doi.org/10.2210/pdb9CT4/pdb] and EMD-45898 [https://www.ebi.ac.uk/emdb/EMD-45898] (diABZI-3 Curved), 9CT6 [https://doi.org/10.2210/pdb9CT6/pdb] and EMD-45900 [https://www.ebi.ac.uk/emdb/EMD-45900] (diABZI-3 Apart), and 9CT5 [https://doi.org/10.2210/pdb9CT5/pdb] and EMD-45899 [https://www.ebi.ac.uk/emdb/EMD-45899] (diABZI-3 Together). Source data are provided with this paper.

Research involving human participants, their data, or biological material

Policy information about studies with human participants or human data. See also policy information about sex, gender (identity/presentation), and sexual orientation and race, ethnicity and racism.

Reporting on sex and gender	n/a
Reporting on race, ethnicity, or other socially relevant groupings	n/a
Population characteristics	n/a
Recruitment	n/a
Ethics oversight	n/a

Note that full information on the approval of the study protocol must also be provided in the manuscript.

Field-specific reporting

Please select the one below that is the best fit for your research. If you are not sure, read the appropriate sections before making your selection.

[x] Life sciences [ ] Behavioural & social sciences [ ] Ecological, evolutionary & environmental sciences

For a reference copy of the document with all sections, see nature.com/documents/nr-reporting-summary-flat.pdf

Life sciences study design

All studies must disclose on these points even when the disclosure is negative.

Sample size	Based on the observed variability (standard deviation) for the neutral control, we determined that n=3 technical replicates were sufficient for a 95% CI. For phospho-STING western experiments, conclusions were drawn from a minimum of 3 independent experiments.
Data exclusions	no data were excluded
Replication	All cell-based experiments are representative of at least n=3 independent experiments
Randomization	not applicable
Blinding	not applicable

Reporting for specific materials, systems and methods

We require information from authors about some types of materials, experimental systems and methods used in many studies. Here, indicate whether each material, system or method listed is relevant to your study. If you are not sure if a list item applies to your research, read the appropriate section before selecting a response.

Materials & experimental systems

n/a	Involved in the study	
[ ]	[x]	Antibodies
[ ]	[x]	Eukaryotic cell lines
[x]	[ ]	Palaeontology and archaeology
[x]	[ ]	Animals and other organisms
[x]	[ ]	Clinical data
[x]	[ ]	Dual use research of concern
[x]	[ ]	Plants

Methods

n/a	Involved in the study	
[x]	[ ]	ChIP-seq
[x]	[ ]	Flow cytometry
[x]	[ ]	MRI-based neuroimaging

Antibodies

Antibodies used	Tubulin (Sigma-Aldrich cat# T5326 1:5000, clone: GTU-88, Lot# 109M4784V), Vinculin (Thermo-Fisher cat# 14-9777-82 1:10,000, clone: 7F9, Lot# 2502412), phospho-STING (Cell Signaling Technology cat# 19781S 1:1000, Lot: 9), STING (Cell Signaling Technology cat# 13647 1:1000, Lot: 3).
Validation	Tubulin: https://www.sigmaaldrich.com/US/en/product/sigma/t5326?srsltid=AfmBOorHbb3pqgGNZts6L5h8bF297-2fTomNzblSdQ52MezD4TcogdHb<br>Vinculin: https://www.thermofisher.com/antibody/product/Vinculin-Antibody-clone-7F9-Monoclonal/14-9777-82<br>phospho-STING: https://www.cellsignal.com/products/primary-antibodies/phospho-sting-ser366-d7c3s-rabbitmab/19781?srsltid=AfmBOooRYPjQPQ7JXJWTYB_ErHoANoLBMaA5pfYzC-2snCrZtgFzec-J<br>STING: https://www.cellsignal.com/products/primary-antibodies/sting-d2p2f-rabbit-mab/13647?srsltid=AfmBOoqXKqna5I8hfdo4Hxn0OUFkS8NcWqPftZ5jbhonxqNZ_EI8oZZ3

Eukaryotic cell lines

Policy information about cell lines and Sex and Gender in Research

Cell line source(s)	InvivoGen cat number #thpl-isg
Authentication	https://www.invivogen.com/thp1-lucia-isg
Mycoplasma contamination	no
Commonly misidentified lines (See ICLAC register)	no

Plants

Seed stocks	n/a
Novel plant genotypes	n/a
Authentication	n/a
