# Supplementary material for: Distinct oligomeric assemblies of STING induced by non-nucleotide agonists
Source: Nat Commun. 2025 Apr 11;16:3440. doi: 10.1038/s41467-025-58641-5 (PMC11992164; doi:10.1038/s41467-025-58641-5)
Supplement: Supplementary file 3 — Description of Additional Supplementary Files [file 41467_2025_58641_MOESM3_ESM.pdf]

File Name: Supplementary Movie 1
Description: 3D Variability Analysis of diABZI-3 bilayer

File Name: Supplementary Movie 2
Description: 3D Variability Analysis of diABZI-3 curved conformation

File Name: Supplementary Data 1
Description: Initial coordinate and simulation input file
